# Editorial: Challenges and innovations in healthcare management and long-term care for an aging society

**DOI:** 10.3389/fmed.2026.1871989

**Published:** 2026-05-21

**Authors:** Lei Qin, Jian Wang

**Affiliations:** 1 School of Statistics, University of International Business and Economics, Beijing, China; 2 Dong Fureng Institute of Economic and Social Development, Wuhan University, Wuhan, China

**Keywords:** health system transformation, healthcare management, healthy aging, long-term care, population aging

Population aging is not merely a demographic transition but a structural transformation that reshapes disease patterns, healthcare systems, and care behaviors. This Research Topic brings together diverse contributions that, when viewed collectively, reveal a coherent multi-level structure of challenges and innovations in healthcare management and long-term care (LTC). Rather than representing isolated findings, these studies jointly point to an integrated framework spanning macro-level disease dynamics, system-level capacity, institutional innovation, and individual behavioral mechanisms.

At the macro level, aging is associated with a rapidly evolving and increasingly complex disease burden. One study demonstrates that the global burden of Alzheimer's disease and related dementias is rising sharply, with strong regional heterogeneity and projections indicating a near doubling by 2050, particularly among the oldest-old and women (Xiang et al.). Another study further shows that liver cancer attributable to drug use in older populations is increasing, driven by the interaction between physiological aging and polypharmacy, reflecting a shift toward more complex, treatment-related risks (Xia et al.). These findings suggest that aging societies face not only higher disease prevalence but also structurally changing risk profiles, requiring dynamic and forward-looking health management strategies.

At the system level, the accessibility and organization of healthcare services play a decisive role in shaping health outcomes. Evidence from this Topic shows that improved access to primary healthcare services significantly reduces health vulnerability among older adults, though inequalities persist across demographic groups (Zhu et al.). Meanwhile, the construction of healthcare data centers enhances health outcomes by enabling data integration, improving service coordination, and promoting digital health management (Yao and Xu). Together, these contributions highlight that both physical access and digital infrastructure constitute foundational components of modern healthcare systems in aging societies.

Moving beyond system capacity, institutional innovation emerges as a critical mechanism for adapting care delivery. A novel “Hospital-at-Nursing-Home” model demonstrates how hospital-level services can be extended into nursing home settings, reducing pressure on hospital beds while enabling more context-sensitive care delivery, albeit with increased clinical risks that require careful management (Ong et al.). In parallel, long-term care insurance (LTCI) is shown to alleviate caregiving burdens and improve household economic outcomes through intergenerational labor supply effects, while also contributing to the reduction of income inequality (Li et al.). These findings illustrate a broader transition from hospital-centered systems toward integrated care systems that combine service innovation with institutional support.

However, system design and institutional arrangements alone are insufficient to ensure effective care utilization. At the micro level, behavioral and psychological mechanisms play a central role in mediating the relationship between healthcare provision and outcomes. One study identifies a persistent gap between older adults' willingness to use community care services and their actual behavior, driven by a combination of enabling, predisposing, and need factors (Yang and Wang). Another study demonstrates that health empowerment significantly enhances self-management among older adults with chronic diseases, both directly and indirectly through resource utilization and self-efficacy (Wang et al.). These findings underscore that healthcare effectiveness depends not only on supply but also on the alignment between services and individual behavioral responses.

Finally, this Research Topic points toward a conceptual shift in how aging itself is understood and managed. The notion of “biological age” is proposed as a more accurate and dynamic measure of health status than chronological age, with important implications for risk assessment, treatment decisions, and personalized care in critical settings (Jiang and Han). This perspective suggests that future healthcare systems must move toward more individualized and data-driven approaches, redefining the boundaries of aging and care.

Synthesizing these contributions, this Research Topic suggests a multi-level framework in which health outcomes in aging societies are jointly determined by: (1) macro-level disease dynamics, (2) system-level access and infrastructure, (3) institutional design and policy arrangements, and (4) individual-level behavioral mechanisms. These dimensions are not independent but interact in complex ways. For example, improvements in healthcare infrastructure may fail to translate into better outcomes if behavioral barriers persist, while institutional innovations such as LTCI can simultaneously influence both care provision and economic wellbeing. Recognizing these interactions is essential for designing effective and sustainable healthcare systems.

In conclusion, addressing the challenges of aging societies requires a shift from fragmented interventions to integrated, multi-level strategies. The contributions in this Research Topic collectively demonstrate that meaningful progress depends on aligning disease surveillance, system capacity, institutional innovation, and human behavior within a coherent framework. Such an approach will be critical for building resilient and equitable healthcare systems in the face of global aging.

